# Efficacy of a cognitive and behavioural psychotherapy applied by primary care psychologists in patients with mixed anxiety-depressive disorder: a research protocol

**DOI:** 10.1186/s12875-015-0248-3

**Published:** 2015-03-20

**Authors:** Amale Jauregui, Joaquín Ponte, Monika Salgueiro, Saloa Unanue, Carmen Donaire, Maria Cruz Gómez, Natalia Burgos-Alonso, Gonzalo Grandes, representing the PSICCAPAD group

**Affiliations:** Sopela Health Centre, Basque Health Service (Osakidetza), Sopela, Spain; Ortuella Mental Health Service, Ortuella, Spain; Primary Care Research Unit of Bizkaia (UIAPB)- Osakidetza, Luis Power 18, 4ª planta, E-48014 Bilbao, Spain; Basic Psychological Processes and Development Department, Faculty of Psychology, University of the Basque Country (UPV/EHU), Donostia-San Sebastian, Spain; School of Nursing, University of the Basque Country (UPV/EHU), Leioa, Spain; Rontegui Health Centre, Barakaldo, Spain; Medical Section, I + D department, BIAL, Bilbao, Spain; Department of Preventive Medicine and Public Health, Faculty of Medicine and Dentistry, University of the Basque Country (UPV/EHU), Leioa, Spain

**Keywords:** Anxiety, Depression, Cognitive-behavioural therapy, Primary care

## Abstract

**Background:**

In contrast with the recommendations of clinical practice guidelines, the most common treatment for anxiety and depressive disorders in primary care is pharmacological. The aim of this study is to assess the efficacy of a cognitive-behavioural psychological intervention, delivered by primary care psychologists in patients with mixed anxiety-depressive disorder compared to usual care.

**Methods/Design:**

This is an open-label, multicentre, randomized, and controlled study with two parallel groups. A random sample of 246 patients will be recruited with mild-to-moderate mixed anxiety-depressive disorder, from the target population on the lists of 41 primary care doctors. Patients will be randomly assigned to the intervention group, who will receive standardised cognitive-behavioural therapy delivered by psychologists together with usual care, or to a control group, who will receive usual care alone.

The cognitive-behavioural therapy intervention is composed of eight individual 60-minute face-to face sessions conducted in eight consecutive weeks. A follow-up session will be conducted over the telephone, for reinforcement or referral as appropriate, 6 months after the intervention, as required.

The primary outcome variable will be the change in scores on the Short Form-36 General Health Survey. We will also measure the change in the frequency and intensity of anxiety symptoms (State-Trait Anxiety Inventory) and depression (Beck Depression Inventory) at baseline, and 3, 6 and 12 months later. Additionally, we will collect information on the use of drugs and health care services.

**Discussion:**

The aim of this study is to assess the efficacy of a primary care-based cognitive-behavioural psychological intervention in patients with mixed anxiety-depressive disorder. The international scientific evidence has demonstrated the need for psychologists in primary care. However, given the differences between health policies and health services, it is important to test the effect of these psychological interventions in our geographical setting.

**Trial registration:**

NCT01907035 (July 22, 2013).

## Background

The prevalence of mental disorders in primary care (PC) in Europe is around 20-55% [1-3]. In association with the current economic crisis, there has been an alarming increase of PC consultations for psychological distress [4,5]. It has been estimated that a quarter of patients who attend PC consultations have had a mental disorder in the previous year, in particular problems related to anxiety and/or depression [1,2,6,7]. In our multi-tiered public health system, around 85-90% of these patients are treated in PC settings [8].

Among mood disorders, the coexistence of anxiety and depression is the norm rather than the exception [9]. Specifically, adjustment disorders are very common, that is, signs and symptoms of mixed anxiety/depression in the context of changing situations that force people to adjust to new situations [1,10].

Clinical practice guidelines recommend cognitive-behavioural therapy (CBT) as the treatment of choice for affective and mood disorders. The use of psychoactive drugs is only recommended for the most severe cases and always in combination with psychological treatment [11-14]. However, the real adherence to these evidence-based clinical guidelines is very poor in general, and several studies have shown that in PC psychoactive drugs are more widely used than psychological techniques, [15]. In relation of this, the Strategy for Mental Health of the Spanish National Health Service recognised that that there has been a significant medicalization of everyday life and that there is a tendency towards an overuse of pharmacological approaches, these requiring less time, as well as less professional expertise and involvement, for the treatment of disorders that should be addressed with specific psychological interventions [16].

There is strong evidence that the efficacy of psychological therapy and CBT in particular, is the same as or greater than pharmacological treatments of the most common affective and anxiety disorders, as well as it having greater long-term effectiveness [17-19]. Notably, the benefits of psychological approaches include a reduction in symptoms associated with anxiety and depression, a decrease in the risk of relapses, the stability of the effect of the treatment in the long term, and high rates of recovery, preventing the condition becoming chronic and decreasing healthcare costs, in terms of medical consultations and use of drugs, as well as sick leave [20-22]. Various studies have suggested that the introduction of psychological interventions in primary care may significantly reduce healthcare and social costs related to mental disorders [23,24]. Specifically, although the cost of psychological treatments may seem high due to the need for trained psychologists, psychological interventions, and CBT in particular, have been found to be more cost-effective in the long term compared to the cumulative costs of the use of antidepressants [25].

The introduction of this type of therapy in routine primary care practice may not be feasible due to, among other reasons, the limited time available per consultation. Some authors have suggested that the role of non-specialist psychologist (although trained in health psychology) should be included in primary care services [26,27]. The objective of this study is to assess the efficacy of CBT-based psychological interventions delivered by primary care psychologists in patients with mixed anxiety-depressive disorder.

### Aims of the study

The primary objective of this study is to assess the efficacy of a CBT-based psychological intervention delivered by psychologists in collaboration with general practitioners (GPs) in primary care settings in patients with mild-to-moderate mixed anxiety-depressive disorder, in terms of perceived quality of life and general health, compared to usual care, provided by GPs.

The secondary objectives are to assess the effect of the CBT-based psychological intervention on the level of anxiety and depressive symptoms, healthcare costs in terms of consumption of psychoactive drugs and use of healthcare resources, and the sick leave taken (number of times and the length of absence), compared to usual care. Additionally, our aim is also to assess the level of satisfaction of doctors and patients in both groups regarding the treatment administered, twelve months after the beginning of the intervention.

### Study hypotheses

The main working hypothesis is that the CBT delivered by psychologists will be more effective than the usual care provided by primary care doctors, in terms of an improvement by at least 10 points on the Short Form-36 General Health Survey (SF-36) in the mid (6 months) and long (12 months) term in patients with mixed anxiety-depressive disorder.

Additionally, the intervention will be more effective than usual care in the reduction of anxiety and depressive symptoms, as assessed by the State-Trait Anxiety Inventory (STAI) and the Beck Depression Inventory (BDI), respectively.

Further, we expect to observe a greater reduction in healthcare costs in terms of consumption of psychoactive drugs and use of healthcare resources as well as in the number and length of sick leave absences in the intervention group than in the control group.

Finally, we also expect GPs and patients to be more satisfied with the intervention than the control treatment.

## Methods/Design

### Design of the trial

This will be an open-label parallel-group randomised controlled trial. Eligible patients will be randomly assigned to one of the study groups: the intervention group (IG), receiving a CBT-based psychological treatment together with usual care, or the control Group (CG) receiving usual care provided by GPs. The principal outcome measure will be the change in quality of life in patients with mixed anxiety-depressive disorder, assessed at four times points: at baseline, that is, pre-treatment; and at 3, 6 and 12 months after the initial assessment.

### Setting

A total of 41 GPs of 7 primary care health centres will participate in the study. The psychologists in charge of delivering the psychological therapy are staff of the Basque Health Service. Patient assessments will be performed by a researcher contracted for the purpose with specific training for taking the required measurements.

The health centres have the necessary infrastructure and resources to carry out the measurements and the intervention.

### Participants and recruitment process

**Figure 1:**
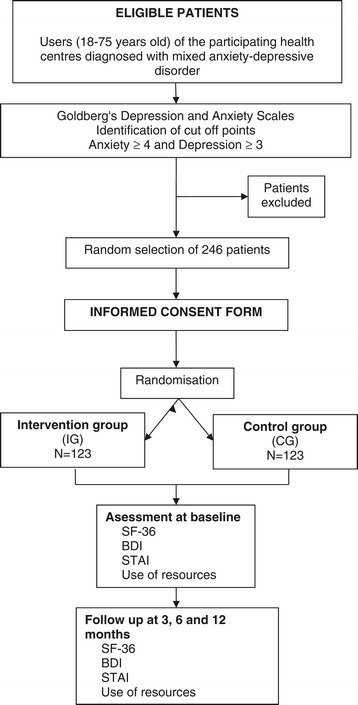
**Flow chart of patient recruitment and randomisation.**

Eligible subjects will be: patients of the participating health centres, over 18 years of age, who have been diagnosed by their GP with mild-to-moderate mixed anxiety-depressive disorder, and obtained scores of at least 4 on the anxiety subscale and at least 3 on the depression subscale of the Goldberg Anxiety and Depression Scale (GADS) [28,29] (see Figure 1 for an overview). We will apply the following exclusion criteria: being over 75 years old; not being able to understand and/or speak Spanish; or having cognitive impairment that could interfere with assessments or intervention; a serious disease or medical condition that could result in death in the following 12 months or a high probability of loss to follow up; or a psychotic or other severe mental illness, as well as a history of suicide attempts or persistent suicidal ideation. Patients who referred to specialised care and any under psychological treatment in the private sector will also be excluded.

The study will be presented to the GPs of the participating centres. Among those interested in participating in the study, we will randomly select (using a computer program) a sample of 41 GPs balanced by health centre.

Each participating GP will be given a list of patients based on the search criterion “active mixed anxiety-depressive disorder”. From each list of eligible patients, a sample of six patients we be randomly selected to be given an appointment at which their GP will explain the details of the study and check that patient meets the inclusion criteria. If they do, the patient will be formally invited to participate, giving them a copy of the patient information leaflet and informed consent form.

Once they have signed the informed consent form, participating patients will be randomly assigned to the IG or the CG following a simple randomisation with a 1:1 allocation ratio using the EpiData 4.0 computer program, with the interviewer and the person in charge of data analysis being blind to group allocation at all times.

### Materials

GPs will use the Spanish version of GADS [28,29] for screening their samples of patients. This scale consists on a short interview questionnaire that has been shown to be useful in the identification of likely cases of anxiety and depression. It has good psychometric properties, with a sensitivity and specificity above 80% [29]. Scores of at least 4 on the anxiety subscale and of at least 3 on the depression subscale have been established as an inclusion criterion.

An assessor trained for the purpose, contracted for the project and blind to patient group allocation, will carry out assessments at four time points over course of the study: baseline (pre-treatment) and 3, 6, and 12 months after the intervention. To assess the level of depression, we will use the BDI [30] validated for the Spanish population by Sanz et al. [31]. The BDI has acceptable psychometric properties as an assessment instrument for measuring depressive symptoms in adults, and is the most widely used questionnaire for assessing the response to treatment of patients with depression.

As for assessing the level of anxiety, we will use the Spanish version of the STAI [32,33]. The STAI is a self-administered questionnaire composed of 40 items rated on a 4-point Likert scale. It assesses two independent types of anxiety: anxiety as a trait (A/T), which refers to relatively permanent and stable dimension of personality or a tendency towards reacting anxiously; and anxiety as a state (A/S), which detects behaviours associated with transient situational anxiety, present during the assessment.

For assessing health-related quality of life and general status, we will use the Spanish version of the SF-36 [34,35]. It is composed of 36 items rated on Likert scales and refers to various aspects of health divided into 8 dimensions (physical functioning, role-physical, bodily pain, general health, vitality, social functioning, role-emotional and mental health). In addition, two global subscores can be calculated that represent the physical and mental state of health: the physical component summary (PCS) and the mental component summary (MCS). All the raw scores of the SF-36 are normalised and transformed to a scale from a 0 to 100, with higher scores indicating better general health status and self-perceived quality of life.

Additionally, at the first (baseline) and the last (12 months) assessments, patients will complete the Health Resource Use Questionnaire, prepared *ad hoc* to collect data on the use of healthcare resources in the previous weeks, as well as the use of drugs and medical consultations, among other factors. Finally, in the last assessment (12 months), all the patients and GPs participating in the study will be asked about their level of satisfaction with the treatment provided, rating it on a visual analogue scale (VAS) ranging from 0 to 10.

### Intervention

Patients assigned to the CG will be treated by their GP and will receive the usual care. Patients assigned to the IG will be contacted by telephone by the psychologist to make a first appointment and plan the subsequent sessions. The design of the intervention is based on CBT programmes for groups developed by local experts in the field [36,37]. The research team has updated these programmes and adapted them for use with individuals, with additional information technology support and giving a more active role to patients.

The intervention consists of 8 individual 60-minute face-to-face sessions, to be carried out during 8 consecutive weeks in the health centres. Six months after the start of the intervention, at the same time as the third measurement, there will be a follow-up session over the telephone, for reinforcement or referral, as appropriate.

Each of the sessions has specific objectives and is focused on various areas:

- 1^st^ session: Psychoeducational explanation of the nature and characteristics of mixed anxiety-depressive disorder. Contextualisation of the intervention. Provision of a copy of the patient handbook.
- 2^nd^ session: Establishment of a programme of rewarding or reinforcing factors based on carrying out regular enjoyable activities, to be recorded by patients in their handbook. Training in relaxation and controlled breathing. Sleep hygiene advice.
- 3^rd^ session: Tackling the symptoms of anxiety. Construction of a hierarchy of feared situations, counselling on exposure to stress factors or feared situations.
- 4^th^ session: Learning and developing techniques focused on detecting and blocking automatic negative thoughts
- 5^th^ session: Learning and developing techniques focused on restructuring automatic negative thoughts
- 6^th^ session: Role playing. Work on situations that perceived as difficult. Learning strategies for problem solving. Self-instruction and self-control.
- 7^th^ session: Developing social skills. Assertiveness and self-concept.
- 8^th^ session: Assessment and end of the intervention. Implementation of strategies to prevent relapses.
- Follow-up session. Telephone consultation lasting for 15 minutes, 6 months after starting the intervention. Identification of high-risk cases; monitoring, reinforcement or referral, as appropriate.

As well as this CBT-based intervention, patients assigned to the IG will also be treated by their GP with the usual care.

### Adverse effects

There is no evidence concerning the potential adverse effects of the CBT-based psychological intervention. However, during the intervention, the psychologist and GP will record any incidents that could be harmful to the patient, regardless of whether there is a causal relationship with the intervention. All the adverse effects will be recorded in a database created for the purpose, stating their severity and causal association with the treatment. Additionally, there will be a committee, independent of the research team that will monitor the safety of the treatment under study and will analyse and extensively review any adverse effects that occur during the study.

### Data quality and management

We have taken various steps to ensure the quality and reliability of the data collected in the study including:

- Creation a specific case report form, with notes on each case
- Double data entry to minimise errors during data storage and processing
- Preparation of documents to support health professionals in decision making and to facilitate the standardisation of the intervention and data collection

### Sample size

We have estimated a target sample of 246 patients, equally divided between the IG and the CG. This sample size would provide a statistical power above 80% for detecting a difference between the IG and the CG of at least 10 points in the overall SF-36 score as significant (p < 0.05) using a two-tailed Student’s t-test (standard deviation = 13.15, obtained from previous study with similar populations [1]). Similarly, this sample size would provide a statistical power above 80% for detecting as statistically significant a difference of at least 10 points in scores on the following dimensions: general health, vitality and mental health in the SF-36 health survey (SD = 22.10).

We have estimated a loss to follow-up of 10% during the 12-month follow-up.

### Statistical analyses

The statistical analysis of data will be performed on an intention-to-treat basis, comparing the changes in the two groups, IG and CG, in the measurements taken 3, 6 and 12 after the initial assessment. The effect attributable to the intervention will be assessed by analysing the difference in the changes between the groups, and 95% confidence intervals will be calculated, adjusting the values for the baseline levels, using analysis of covariance (ANCOVA). As the baseline level, results will be adjusted for covariable that could confound or modify the effect of the intervention.

To take into account the potential effect of having patients grouped by GPs, the aforementioned ANCOVA models will be extended to mixed-effect models that include the random effect of each GP, both on the intercept and the effect of the intervention.

Finally, to analyse the overall changes in health-related quality of life over the 12 months of the study, the effect of time on the 4 repeated measures collected from each patient will be estimated using a longitudinal mixed-effect model with fixed effects (intervention, time, time-intervention interaction) and random effects (specific effect of each subject and each doctor and health centre).

Interactions between time and the specific effect of subjects, GPs and health centres will also be explored.

Data analysis will be performed using the statistical package SAS for Windows.

### Legal and ethical considerations

This study complies with the Declaration of Helsinki and its subsequent reviews, as well as with the Good Clinical Practice guidelines. We have taken measures for safeguarding the confidentiality of the information collected. Only the researchers participating in the study will have access to the data related to patients.

The present project has been approved by the Clinical Research Ethics Committee of the Basque Country (CEIC-E). In addition, the legal representative of each health centre has signed a document stating that they understand the study and are committed to collaborate with the services involved.

### Limitations

Patients will be selected retrospectively based on their medical diagnosis, and hence patients with mixed anxiety-depressive disorder who have not been given this diagnosis or whose diagnosis has not been coded in their medical record will not be included in this study, affecting the external validity of the study.

The assessor and initially the GPs will be blind to group allocation. However, in our design, it is not possible for patients to be masked to treatment, and therefore we are not able control the interaction between patients and GPs.

This study has also a feasibility issue, given that it attempts to explore changes in patients after an intervention delivered by health professionals who are not currently present in primary care, that is, psychologists. Hence, it is possible that we will fail to recruit the number of psychologists required to work with the sample of patients.

## Discussion

The prevalence rates of mental health conditions and in particular, anxiety and depression have shown an upward trend in recent years, probably exacerbated by the current economic situation and high rates of employment in our setting [38]. According to the results of the latest Spanish Health Survey, it is estimated that around 15% of the adult population has some type of mood disorder related to anxiety and depression [39], a much high percentage than in previous years. Since 2006, we have observed significant growth in the percentage of patients seeking medical assistance for psychological problems [4]. Further, the impact of mental disorders on quality of life and general health is even greater than that of other chronic disorders, such as arthritis, diabetes, cardiovascular or respiratory diseases [40], and is often associated with a significant increase in the use of healthcare resources [41,42]. Mental health conditions account for approximately 20% of health expenditure in developed countries, and this percentage is expected to rise in the near future.

According to the World Health Organisation, despite the resources dedicated to mental healthcare in terms of prescriptions of psychoactive drugs, medical consultations, and emergency services, as well as the costs of related work absenteeism and sick leave, most patients with mental health problems do not receive the best treatment for their condition [40]. The key evidence-based clinical practice guidelines recommend CBT-based psychological interventions for treating mild-to-moderate, subclinical mood disorders, and recommend against routinely prescribing anxiolytics and antidepressants [12,13].

Most research on the effectiveness of CBT for the treatment of emotional disorders has focused on specialised mental care. However, in our health service, most patients with non-severe mood disorders are treated in primary care and are not referred to mental health specialists [41]. In general, patients themselves prefer to be treated in the primary care setting, it been more accessible and less stigmatised than mental health services [43]. However, the treatment of psychological problems in primary care is currently still over-medicalised, based on a traditional biomedical model, in which the prescription of antidepressants is the treatment of choice in most cases. In this context, the introduction of psychological care services in primary care seem a necessary step towards a biopsychosocial model, in which mental health problems are tackled in an integrated way.

There have been some attempts to train primary care health professionals, in particular doctors and nurses, in psychological counselling techniques for addressing the problems related to the management of stress, anxiety and depression. However, this type of training does not seem to result in benefits over the usual care provided in primary care [44]. It seems clear that the most suitable professionals to carry out CBT interventions are psychologists, who have a specific training in the diagnosis and treatment of mental health conditions and could work at the primary care level.

The aim of this clinical trial is to assess the efficacy of a CBT-based psychological intervention, delivered by psychologists at the PC level, in patients with mixed anxiety-depressive disorder, compared to the usual care provided by their GPs. Although some studies in other places have provided promising results, differences in health policies and public health systems between countries make it necessary to assess the effectiveness of psychological interventions in our geographical setting. We also want to evaluate their potential effect on the reductions in health expenditure in terms of consumption of psychoactive drugs, medical consultations, and emergency department attendances, as well as in work absenteeism and sick leave.

If the results of this study were to be in line with the expectations of the research team, health authorities should roll out this type of intervention. This would involve an organisational and structural change at very timely moment in the current process of integration between PC and specialised care in our health care system.

## Abbreviations

BDI: Beck Depression Inventory
CBT: Cognitive-behavioural therapy
CG: Control group
CRF: Case report form
GP: General practitioner
IG: Intervention group
PC: Primary care
SF-36: SF-36 Health Survey
STAI: State-Trait Anxiety Inventory
